# Echinacea and elderberry—should they be used against upper respiratory tract infections during pregnancy?

**DOI:** 10.3389/fphar.2014.00031

**Published:** 2014-03-04

**Authors:** Lone Holst, Gro C. Havnen, Hedvig Nordeng

**Affiliations:** ^1^Department of Global Public Health and Primary Care and Centre for Pharmacy, Faculty of Medicine and Dentistry, University of BergenBergen, Norway; ^2^Regional Medicines Information and Pharmacovigilance Centre (RELIS), Oslo University HospitalOslo, Norway; ^3^Department of Pharmacy, Faculty of Mathematics and Natural Sciences, School of Pharmacy, University of OsloOslo, Norway

**Keywords:** Echinacea, Elderberry, pregnancy, safety, efficacy, CAM, respiratory infection

## Abstract

This review evaluates the safety of echinacea and elderberry in pregnancy. Both herbs are commonly used to prevent or treat upper respiratory tract infections (URTIs) and surveys have shown that they are also used by pregnant women. The electronic databases PubMed, ISI Web of Science, AMED, EMBASE, Natural Medicines Comprehensive Database, and Cochrane Library were searched from inception to November 2013. Relevant references from the acquired articles were included. No clinical trials concerning safety of either herb in pregnancy were identified. One prospective human study and two small animal studies of safety of echinacea in pregnancy were identified. No animal- or human studies of safety of elderberry in pregnancy were identified. Twenty clinical trials concerning efficacy of various echinacea preparations in various groups of the population were identified between 1995 and 2013. Three clinical trials concerning efficacy of two different elderberry preparations were identified between 1995 and 2013. The results from the human and animal studies of *Echinacea* sp. are not sufficient to conclude on the safety in pregnancy. The prospective, controlled study in humans found no increase in risk of major malformations. The efficacy of *Echinacea* sp. is dubious based on the identified studies. Over 2000 persons were given the treatment, but equal amounts of studies of good quality found positive and negative results. All three clinical trials of Elderberry concluded that it is effective against influenza, but only 77 persons were given the treatment. Due to lack of evidence of efficacy and safety, health care personnel should not advice pregnant women to use echinacea or elderberry against upper respiratory tract infection.

## Introduction

This review considers two herbal treatments against upper respiratory tract infection (URTI); *Echinacea* sp. and *Sambucus nigra* and the safety of their use by pregnant women. *Echinacea* sp. are commonly used by pregnant women (Hepner et al., 2002; Nordeng and Havnen, 2004; Holst et al., 2009a; Heitmann et al., 2010), but documentation of safety in pregnancy is sparse. *Sambucus nigra* is used by pregnant women in Norway (Nordeng and Havnen, 2004) and the USA (Tsui et al., 2001) while no documentation of use in other regions is available to our knowledge. The use of herbal remedies among pregnant women in the western world is common though the documentation of safety and efficacy is lacking (Nordeng and Havnen, 2004; Forster et al., 2006; Lapi et al., 2008; Holst et al., 2009a; Cuzzolin et al., 2010; Facchinetti et al., 2012). Clinical trials of herbs are not common and for ethical reasons pregnant women are so far only included in trials of herbs against pregnancy-specific conditions like nausea and vomiting (NVP) (Pongrojpaw et al., 2007; Ensiyeh and Sakineh, 2008; Ozgoli et al., 2009). Still pregnant women use herbs against many other conditions (Nordeng and Havnen, 2004; Holst et al., 2009a; Heitmann et al., 2010).

Pharmaceuticals are generally not tested in pregnant women, but the drug substances are tested for their teratogenic potential in two animal species before they are approved for human use. Whether this gives a good prediction of teratogenic potential in humans, is controversial, but in many cases it gives an indication to be followed up by pharmacovigilance (Koren and Nordeng, 2013). New teratogenic effects are often first reported as case reports. These can be followed by observational studies of exposed pregnant women compared to healthy pregnant controls or disease matched women. Linking of various registries like a prescription registry with the medical birth registry can give us important information about teratogenicity of pharmaceuticals. Herbal remedies are not prescribed and their use is therefore not registered. Only the user has the information and if she is not asked by health care personnel in antenatal care or does not reveal her herb use, no link between herbs and pregnancy outcome can be made. In large observational studies like the Norwegian Mother and Child Cohort study (Norwegian Institute of Public Health, 2007) the safety of commonly used herbs can be studied (Heitmann et al., 2013), but even studies like that of more than 100.000 pregnancies may be limited by study power if the frequency of herbal use is low as malformations rarely occur and most teratogens cause only a moderate rise in risk. Heitmann et al. (2013) found that their study had ≥80% statistical power to rule out a doubling or more of the risk of major malformations after consumption of ginger (*n* = 466 in the first trimester) during early pregnancy.

Herbs can pose various risks in pregnancy (Schaefer et al., 2007). Some herbs like black cohosh (*Actaea racemosa* L.) or blue cohosh (*Caulophyllum thalictroides* (L.) Michaux) have traditionally been used to stimulate menstruation or provoke abortion. Alkaloid-containing herbs like barberry (*Berberis vulgaris* L.) are potentially hepatotoxic, but many of them are used by medical herbalists to treat conditions like constipation or heartburn. Laxatives containing anthraquinones from for instance senna (*Senna alexandrina* Mill.) or cascara (*Rhamnus purshiana* DC.) are effective stimulants of the bowel peristalsis, but might theoretically also stimulate uterus. Some women might substitute necessary prescribed pharmaceuticals for herbs due to a belief in their safety and will thus not be treated properly for a serious condition. Others might use a herbal product before they become pregnant and just continue the use unconsciously. Some herbal products have been found to be contaminated with heavy metals or deliberately added pharmaceuticals and in some cases misidentified herbs have been included (Schaefer et al., 2007).

A benefit-risk evaluation is essential when a pregnant woman considers using a herbal product. The fact that there is no documentation of safety does not mean that there is a risk—it just means that we don't know. If the benefit is substantial, it might be reasonable to use the product in spite of the sparse safety documentation. This is commonly the case for pharmaceuticals. The use of for instance antiepileptic drugs is essential for the mother and although the drug may pose a risk for the fetus, the benefit in many cases is found to outweigh the risk. As the use of herbal products is hardly essential (at least in the western world) the risk should preferably be documented to be minute before a product is recommended.

*Echinacea* sp. used for treatment of URTI (mainly cold) are *Echinacea purpurea, Echinacea pallida*, and *Echinacea angustifolia*. Used plant parts are “herba” and “radix,” separately or combined. The remedies are manufactured by different extraction methods possibly leading to extraction of different constituents and/or different amounts of the constituents. The herbal remedies are sold as tablets, tincture, or tea. For those reasons it is difficult to compare herbal remedies containing *Echinacea* sp. The European Medicines Agency, EMA, has developed monographs for *Echinacea purpurea* herba and radix, *Echinacea pallida* radix and *Echinacea angustifolia* radix (European Medicines Agency, 2008, 2009, 2010, 2012). In accordance with these legal texts none of the licensed herbal products containing *Echinacea* sp. should be used during pregnancy or lactation due to lack of sufficient data. The only exception is topical use of *Echinacea purpurea* herba on other areas than the breast, this because systemic absorption is not expected. The German commission E Monographs on the other hand state that no restrictions on use during pregnancy or lactation are known except from parenteral use of *Echinacea purpurea* root (Blumenthal et al., 2000), however no references to scientific papers are given.

*Sambucus nigra* berry is used for treatment of URTI, mainly the flu. The European Medicines Agency, EMA, has worked on a monograph for the berries, but has terminated the work in March 2013 due to lack of information on traditional use with a specified dosage for at least 30 years (including 15 years in the EU) (European Medicines Agency, 2013a,b). Due to lack of information they do not recommend use of the berries during pregnancy or lactation.

Importantly, many trademark products containing echinacea or elderberry will be defined as dietary supplements and thus not be legally bound to follow the recommendations in the official plant monographs.

The aim of this study was to review the literature on safety during pregnancy and efficacy against URTI of *Echinacea* sp. and *Sambucus nigra* to help health care personnel to make evidence based decisions about their recommendations and advice.

## Materials and methods

### Data sources

The electronic databases PubMed, ISI Web of Science, AMED, EMBASE, Natural Medicines Comprehensive Database, and Cochrane Library were searched from inception to November 2013 and relevant references from the acquired articles were also included. The applied search words/terms were:

- Safety/reproductive toxicology AND pregnant/pregnancy AND *Echinacea*/coneflower
- Safety/reproductive toxicology AND pregnant/pregnancy AND *Sambucus nigra*/elderberry
- Efficacy AND *Echinacea*/coneflower
- Efficacy AND *Sambucus nigra* /elderberry

*Echinacea* sp. covers *Echinacea purpurea, Echinacea pallida*, and *Echinacea angustifolia*.

### Data extraction

Acquired references were handled according to PRISMA 2009 flow diagram (Moher et al., 2009). This states four steps:

1. Identification: number of records identified through database searching and number of additional records identified through other sources
2. Screening: number of records after duplicates removed leading to number of records screened again leading to number of records excluded and
3. Eligibility: number of full text articles assessed for eligibility leading to number of full text articles excluded (with reason) and
4. Included: number of studies included in the qualitative synthesis leading to number of studies included in the quantitative synthesis

This analysis was performed for the searches A–D separately.

### Study selection

Articles excluded:

- other languages than English and the Scandinavian languages
- multi-herbal products
- *in vitro* studies
- other diagnoses than cold/flu (with respect to efficacy)
- Articles more than 20 years old (from 2013)
- Articles reporting prevalence of drug use
- Reviews
- Conference abstracts, letters, notes, editorials

The quality of the studies was evaluated according to criteria from the Cochrane Handbook for Systematic Reviews of Interventions (Higgins and Green, 2011).

## Results

**Figure 1:**
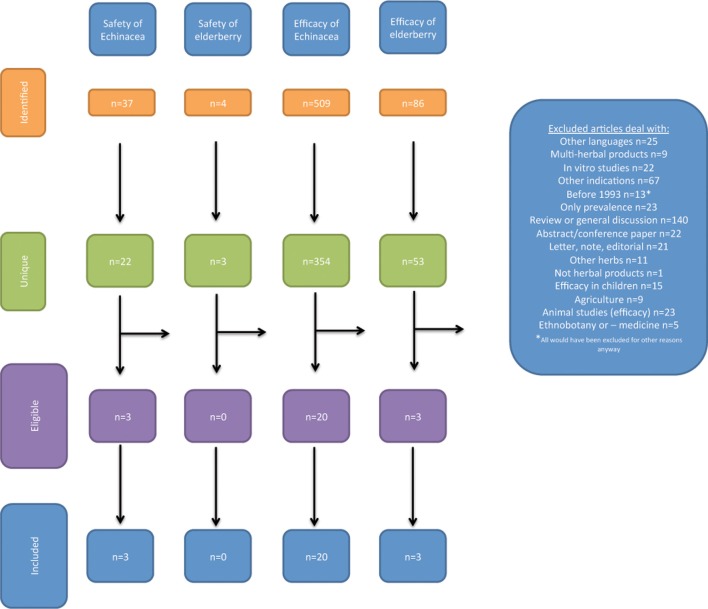
**Identification of the studies included in the review**.

The studies revealed in the literature searches and the selection of studies for the review is illustrated in Figure 1.

### *Echinacea sp*.

The only available human data on safety of echinacea in pregnancy come from one study of 412 pregnant women whereof 206 had used echinacea as tablets or tincture in various doses and with the most common duration being 5–7 days (Gallo et al., 2000). This cohort was disease-matched to women exposed to non-teratogenic agents by maternal age, alcohol intake and smoking, and rates of major and minor malformations were compared. No statistically significant differences were found between the groups with respect to pregnancy outcome, delivery method, maternal weight gain, gestational age, birth weight, fetal distress, or major malformations. See Table 1. No case reports describing side effects were located.

**Table 1:** **Clinical trials and other human and animal studies of safety of *Echinacea* sp. in pregnancy**.

**Author and year of publication**	**Description**	**Conclusion**
***Echinacea* sp. (*Echinacea purpurea, pallida*, and/or *angustifolia*. Products have various combinations of those three herbs or just one or two.)**		
Gallo et al. (2000)	Doses: Tablets 250–1000 mg/day Tincture 5–30 drops/day	No statistically significant difference in terms of pregnancy outcome (gestational age, birth weight, fetal distress or minor or major malformations), delivery method or maternal weight gain
Prospective study	Duration: most commonly 5–7 days	
HUMAN	Participants (treatment:control): 206:206	
Chow et al. (2006)	Doses: 0.45 mg/day/body weight	Cell types (splenic lymphocytes and nucleated erythroid cells) normally increased during pregnancy were significantly reduced in *E. purpurea*-consuming mice to the level of non-pregnant mice. Bone marrow was not influenced by *E. purpurea* Increased risk of early fetal resorption. Fetal resorption was seen as pregnant mice on diet without *E. purpurea* had a mean of 4.7 fetuses at 10–11 days and 4.0 at 12–14 days while mice on diet with *E. purpurea* had 4.0 and 2.0, respectively The authors argue that extrapolation to humans may not be unreasonable and indicate that *E. purpurea* can cause spontaneous abortion
ANIMAL	Six pregnant mice were given a controlled diet with echinacea and seven were given a diet without Eight non-pregnant mice were given a diet without Three mice from each group were euthanized at the gestational ages 10–11 and 12–14 days. Fetal status (dead/alive) and number was registered. Number of hemopoietic cells from spleen and bone marrow from the pregnant mice was counted	
Barcz et al. (2007)	Eight pregnant mice were given one of three different brands of *E. purpurea* tablets dissolved in water 0.6 mg/day/body weight; four mice were give water as controls from day 1 in pregnancy. Euthanized on the 18th day of pregnancy, embryos extracted and weighted. Embryos from one litter homogenized for testing for angiogenesis and cytokine levels	The various *E. purpurea* products gave contradictive results with respect to angiogenesis. Cytokine level was lower in all treated animals than in controls. Number of fetuses in one litter was slightly (but not significantly) lower after treatment with two of the three *E. purpurea* products compared to the third and control. The authors found that *E. purpurea* may influence fetal angiogenesis in mice and thus should not be recommended to pregnant women
ANIMAL		

Two animal studies on reproductivity are also available (Chow et al., 2006; Barcz et al., 2007). See Table 1.

Twenty studies of the efficacy of *Echinacea* sp. against URTI were identified; see Table 2 (Dorn et al., 1997; Hoheisel et al., 1997; Melchart et al., 1998; Brinkeborn et al., 1999; Grimm and Muller, 1999; Lindenmuth and Lindenmuth, 2000; Turner et al., 2000; Schulten et al., 2001; Barrett et al., 2002, 2010; Schwarz et al., 2002; Goel et al., 2004, 2005; Sperber et al., 2004; Yale and Liu, 2004; Turner et al., 2005; Schoop et al., 2006; Hall et al., 2007; O'Neil et al., 2008; Jawad et al., 2012). Doses are difficult to compare as the formulations vary and are described in mg root/herb, mg extract or as “a standardized formulation.” One study included only men (Schwarz et al., 2002) and 12 studies specifically excluded pregnant women (Hoheisel et al., 1997; Melchart et al., 1998; Grimm and Muller, 1999; Lindenmuth and Lindenmuth, 2000; Schulten et al., 2001; Barrett et al., 2002, 2010; Goel et al., 2004; Sperber et al., 2004; Yale and Liu, 2004; O'Neil et al., 2008; Jawad et al., 2012). One study was open (Schoop et al., 2006), 19 were randomized, controlled trials (Dorn et al., 1997; Hoheisel et al., 1997; Melchart et al., 1998; Brinkeborn et al., 1999; Grimm and Muller, 1999; Lindenmuth and Lindenmuth, 2000; Turner et al., 2000, 2005; Schulten et al., 2001; Barrett et al., 2002, 2010; Schwarz et al., 2002; Goel et al., 2004, 2005; Sperber et al., 2004; Yale and Liu, 2004; Hall et al., 2007; O'Neil et al., 2008; Jawad et al., 2012). Twelve studies considered treatment of URTI (Dorn et al., 1997; Hoheisel et al., 1997; Brinkeborn et al., 1999; Lindenmuth and Lindenmuth, 2000; Schulten et al., 2001; Barrett et al., 2002, 2010; Goel et al., 2004, 2005; Sperber et al., 2004; Yale and Liu, 2004; O'Neil et al., 2008); one considered only prophylaxis (Schwarz et al., 2002) and seven considered both aspects (Melchart et al., 1998; Grimm and Muller, 1999; Turner et al., 2000, 2005; Schoop et al., 2006; Hall et al., 2007; Jawad et al., 2012). Three studies used viral challenge (Turner et al., 2000, 2005; Sperber et al., 2004) while the rest studied naturally occurring disease.

**Table 2:** **Clinical trials and other human studies on the efficacy of *Echinacea* sp. against upper respiratory tract infections**.

**References**	**Study population**	**Intervention**	**Comparator**	**Outcome**
	**Participants completing the study.**			
	**Given as treatment:control where only two groups and otherwise with letters A, B, C, etc. referring to interventions**			
Jawad et al. (2012)	Healthy individuals, ≥18 years old	Echinaforce^®^ tincture (*E. purpurea*, 95% herba + 5% radix)	Placebo	In the placebo group significantly more days where participants experienced a cold, recurrent infections, cold episodes treated with pain medication and membraneous viruses detected in nasal secretion were registered
UK	Participants: 673 (325:348)	Prevention: 3 × 0.9 ml/day (2400 mg extract)		
RCT	Pregnant and lactating women excluded	Treatment 5 × 0.9 ml/day (4000 mg extract)		
		Started on preventive dose, increased to treatment dose when needed		
Barrett et al. (2010)	Individuals with a cold started within the last 36 h, ≥12 years old	*E. purpurea* root and *E. angustifolia* root	Placebo	Mean global severity and mean illness duration were slightly lower for the two echinacea-groups than for the other two groups, but none of the differences were statistically significant
USA	Participants: 719 (A:174. B:182. C:179. D:184)	Echinacea corresponding to 10.2 g dried root during first 24 h, then 5.1 g each of the next 4 days 4 groups: A: no treatment, B: placebo tablets, C: echinacea tablets blinded, D: echinacea tablets open-label		
RCT	Pregnant women excluded			
O'Neil et al. (2008)	Convenience sample of healthy adults working in a university health care center, 18–65 years old	*E. purpurea* 300 mg capsules, 3 × 2 daily for 8 weeks	Placebo = parsley	No significant difference in number of days with cold symptoms, median number of sick days, or mild adverse effects between the two groups
USA	Participants: 58 (28:30). Pregnant and lactating women excluded			
RCT				
Hall et al. (2007)	Healthy active adults.	*E. purpurea* Nature's Way^®^ capsules 2 × 4 daily	Placebo	No difference was found in the number of colds experienced, but the echinacea-group had a significantly shorter duration of their cold-episodes
USA	Participants: 32 (18:14)			
RCT				
Schoop et al. (2006)	Athletes recruited through GPs or sports physicians, 18–75 years old	Echinaforce forte^®^ 750 mg (*E. purpurea*; 18.6 mg dried plant extracted), 95% herb + 5% radix. 1 × 2 daily for 8 weeks	None	Seventy-one percent of the participants had no cold episodes (symptoms for more than 3 days) during the treatment period, 26% had 1 and 3% had 2 episodes
Switzerland				
Open	Participants: 80			
Turner et al. (2005)	Healthy, University students	*E. angustifolia* radix. 3 different extracts. 1.5 ml tincture (300 mg radix) × 3 daily from day −7 to day +5, viral challenge at day 0 7 groups (3 groups given treatment in both prophylaxis- and treatment phase, 3 given placebo for prophylaxis, 1 given placebo all through)	Placebo	No statistically significant effects were detected: Prophylaxis had no effect on the infection rate after viral challenge. Treatment had no effect on virus titer. Treatment had no effect on symptom score or on proportion of participants with clinical cold. No effect on course of the illness by prophylaxis or treatment. No effect on the amount of nasal secretion. No effect on inflammatory markers
USA	Participants: 399 (52:52:45:48:51:48:103)			
RCT				
Goel et al. (2005)	Adults, 18–65 years old Recruited via advertisements	Echinilin^®^ standardized formulation of *E. purpurea* (various parts) liquid formulation. 8 doses during first 24 h, 3 daily doses for 6 days. 1 dose = 5 ml.	Placebo	Symptom scores were significantly lower than day one-level on day 4 in the treatment group and on day 7 in the placebo group. In the placebo group the score was significantly higher than day one-level on days 2–4 while in the treatment group it never became significantly higher
Canada				
RCT	Participants: 56 (25:31)			
Goel et al. (2004)	Adults, 18–5 years old Recruited via advertisements	Echinilin^®^ standardized formulation of *E. purpurea* (various parts) liquid formulation. 10 doses during first 24 h, 4 daily dose for 6 days	Placebo	Total symptom scores were significantly lower in the treatment group compared to the placebo group throughout the study. All symptoms except cough showed a shorter duration in the treatment group
Canada	Participants: 111 (54:57)			
RCT	Pregnant and lactating women excluded			
Sperber et al. (2004)	Healthy individuals, 18–65 years old	EchinaGuard^®^ juice of *E. purpurea* herb in ethanol, 2.5 ml × 3 daily for 14 days. Virus inoculation after 7 days	Placebo	Infection rate was not decreased by treatment with echinacea before and after inoculation. No significant difference between treatment- and placebo group in amount of persons developing a cold. No significant difference in daily symptom scores
USA	Participants: 46 (24:22)			
RCT	Pregnant and lactating women excluded			
Yale and Liu (2004)	Patients with a cold, ≥18 years old Recruited via advertisements	EchinaFresh^®^ *E. purpurea* herb 100 mg, freeze dried juice (capsules) × 3 daily until symptoms relieved or up to maximum 14 days. Treatment started within the first 24 h of symptoms	Placebo	No statistically significant difference in symptom scores or time to resolution of symptoms between the groups
USA	Participants: 128 (63:65)			
RCT	Pregnant and lactating women excluded			
Schwarz et al. (2002)	Healthy male, 20–40 years old	Esberitox^®^ *E. purpurea* herb ethanol extract × 2 daily for 14 days, cross-over with placebo, 4 weeks wash-out in between	Placebo	Immune stimulatory effects were not seen after oral administration in healthy individuals
Germany	Participants: 40			
RCT				
Barrett et al. (2002)	Patients with a cold, ≥18 years old Recruited via advertisements	Capsules of 1 g dried root and herb of *E. purpurea* and root of *E. angustifolia* six times during first 24 h and 3 times each subsequent day for a maximum of 10 days.	Placebo = alfalfa	No significant difference in severity or duration of cold
USA	Participants: 142 (69:73)			
RCT	Pregnant women excluded			
Schulten et al. (2001)	Patients with a cold, ≥18 years old	Echinacin^®^, *E. purpurea* herb pressed juice, 5 ml × 2 daily for 10 days	Placebo	Number of days with “the complete picture of” common cold was significantly reduced. The cold was experienced as “less severe” in the treatment group
Germany	Recruited via employer (Madaus AG manufacturer of Echinacin^®^) Participants: 70 (37:33)			
RCT	Pregnant and lactating women excluded			
Lindenmuth and Lindenmuth (2000)	Patients with a cold, ≥18 years old	Echinacea Plus^®^ herbal tea. *E. purpurea* and *E. angustifolia* herb + extract of *E. purpurea* root corresponding to 1275 mg dry plant per teabag. Five to six cups the first day and reducing with one cup per day for the next 5 days	Placebo = Eaters Digest^®^ tea	Treatment relieved symptoms of cold/flu significantly more effective than control tea. The symptoms lasted significantly shorter with treatment and the treatment group experienced significantly fewer days of noticeable symptoms
USA	Recruited among employees in a nursing home			
RCT	Participants: 95 (48:47)			
	Pregnant and lactating women excluded			
Turner et al. (2000)	Patients with a cold, ≥18 years old	*E. angustifolia* 300 mg × 3 daily for 14 days before virus challenge, then same treatment for 5 days	Placebo	No significant effect on either the occurrence of infection or the severity of illness
USA	Recruited from a university community			
RCT	Participants: 92 (50:42)			
Brinkeborn et al. (1999)	Healthy individuals, ≥18 years old Recruited via advertisements	A:Echinaforce^®^ (6.78 mg 5% herba and 95% radix crude extract), B:*E. purpurea* herba and radix concentrate (48.27 mg of the same extract) and C:*E. purpurea* radix 29.6 mg crude extract or D:placebo. 2 × 3 daily for no more than 7 days	Placebo	Echinaforce^®^ and the herb and root concentrate both showed significant reductions in “complaint index” compared to placebo according to doctor's record and according to the patient's record
Sweden	Participants: 180 (A:41. B:49. C:44. D:46)			
RCT	Pregnant and lactating women excluded			
Grimm and Muller (1999)	Patients with a cold, ≥12 years old	Echinacin-Liquidum^®^ (fluid extract of *E. purpurea* herba), 4 ml × 2 daily for 8 weeks	Placebo	No significant difference in incidence, duration or severity of colds, and respiratory infections
Germany	Recruited by GP			
RCT	Participants: 101 (50:51)			
	Pregnant and lactating women excluded			
Melchart et al. (1998)	Healthy individuals, ≥18 years old	Ethanolic extract of A:*E. purpurea* root or B:*E. angustifolia* root or C: placebo. 50 drops × 2 daily for 12 weeks from Monday to Friday	Placebo	No significant difference in number, severity, or duration of upper respiratory tract infections, quality of life, time to occurrence of infection, or white blood cell counts
Germany	Recruited via advertisements			
RCT	Participants: 244 (A:84. B:85. C:75)			
	Pregnant women excluded			
Hoheisel et al. (1997)	Patients with a cold, ≥18 years old	Echinagard^®^ (*E. purpurea)* 20 drops every 2 h the first day, then 3 times daily for up to 10 days	Placebo	Significantly fewer participants in the treatment group experienced “fully expressed symptoms of acute respiratory infection” (a “real” cold), but no difference was seen in intensity of symptoms between the groups. Patients in treatment group showed significantly more rapid recovery
Sweden	Recruited by company physician			
RCT	Participants: 120 (60:60)			
	Pregnant and lactating women excluded			
Dorn et al. (1997)	Patients with URTI, ≥18 years old	900 mg *E. pallidae* radix liquid preparation, 8–10 days	Placebo	Duration of illness and symptom scores of cold, weakness, pain in arms and legs, and headache were significantly reduced in the treatment group
Germany	Recruited by GP			
RCT	Participants:160 (80:80)			

Evaluation of the quality of the studies is given in Table 3. “Random sequence generation” and “Allocation concealment” can give an indication of selection bias. Studies with minus or question mark in those columns are at a higher risk of selection bias than those with a plus.

**Table 3:** **Quality of the studies of efficacy of *Echinacea* sp**.

**Paper**	**T/P/B^1^**	**Random sequence generation**	**Allocation concealment**	**Blinding of patient and personnel**	**Recruitment**	**Quality**	**Pos/neg result**
Jawad et al., 2012	B	+	+	+	Advert on university campus	High	Pos
Hall et al., 2007	B	+	+	+	?	High	Pos
Goel et al., 2005	T	+	+	+	Media advert	High	Pos
Goel et al., 2004	T	+	+	+	Media advert	High	Pos
Hoheisel et al., 1997	T	+	+	+	Employees at furniture factory	High	Pos
Dorn et al., 1997	T	+	+	+	GP	High	Pos
Barrett et al., 2010	T	+	+	+	Media advert, e-mail, word of mouth	High	Neg
O'Neil et al., 2008	T	+	+	+	Convenience sample of employees in uni. med. center	High	Neg
Schwarz et al., 2002	P	+	+	+	?	High	Neg
Barrett et al., 2002	T	+	+	+	Posters, newspapers, e-mail	High	Neg
Grimm and Muller, 1999	B	+	+	+	Patients from GP	High	Neg
Schulten et al., 2001	T	+	+	+	Employees at manufacturer	Moderate	Pos
Lindenmuth and Lindenmuth, 2000	T	?	?	+	Employees at nursing home	Moderate	Pos
Brinkeborn et al., 1999	T	+	?	?	Media advert	Moderate	Pos
Turner et al., 2005	B	?	?	+	?	Moderate	Neg
Schoop et al.,^Open^ 2006	B	–	–	–	GP's and sports physicians	Low	Pos
Sperber et al., 2004	T	?	?	?	?	Low	Neg
Yale and Liu, 2004	T	?	?	?	Media advert	Low	Neg
Melchart et al., 1998	B	?	?	?	Military sites and industrial plant, posters and info-events	Low	Neg
Turner et al., 2000	B	–	–	–	?	Low	Neg

Open open study. Others are RCT.

1 Treatment, prevention, both.

?, unclear.

The studies have evaluated the efficacy of *Echinacea* sp. from one or more of the following criteria:

- Number of episodes of cold or during treatment
- Duration of illness
- Additional painkillers or other pharmaceuticals used
- “symptom score”/severity of illness
- Virus count in nasal secretion
- Infection rate after viral challenge

All criteria had approximately as many positive as negative results, but “Infection rate after viral challenge,” described in three studies had only negative results. It was thus not possible to find a reduction in infection rate after virus challenge in patients taking echinacea prophylactics or for treatment of an induced cold.

### *Sambucus nigra*, elderberry

Neither human nor animal studies of the safety of *Sambucus nigra* in pregnancy were identified. Only three relevant studies on the efficacy of *Sambucus nigra* against URTI were identified (Zakay-Rones et al., 1995, 2004; Kong, 2009). All were randomized, controlled trials of various sizes; between 15 and 32 persons were treated with *S. nigra*. See Table 4.

**Table 4:** **Clinical trials and other human studies on the efficacy of *Sambucus nigra* against upper respiratory tract infections**.

**References**	**Study population Participants (treatment:control, where only two groups) completing the study**	**Intervention**	**Comparator**	**Outcome**
Kong (2009)	Patients with flu symptoms, 16–60 years old	ViraBLOC^®^, elderberry extract 175 mg as slow-dissolve lozenges; × 4 daily for 2 days	Placebo	No difference between symptom scores (fever, headache, muscle aches, cough, mucus discharge, and nasal congestion) in treatment and control group at onset of treatment. Significant difference for 4 out of 6 scores at 24 h and for all six at 48 h. Improvement in treatment group and worsening in placebo
China	College students			
RCT	Participants: 64 (32:32)			
	Pregnant and lactating women excluded			
Zakay-Rones et al. (2004)	Patients with flu symptoms, ≥18 years old	Sambucol^®^, 15 ml × 4 daily for 5 days	Placebo	No difference between symptom scores (aches, cough, quality of sleep, mucus discharge, nasal congestion, “global evaluation”) in treatment and control group at onset of treatment. Relief of symptoms came significantly faster in treatment group (day 3–4 vs. day 7–8) and significantly less rescue-medication was used.
Norway	Recruited by GP			
RCT	Participants: 60 (30:30)			
	Pregnant and lactating women excluded			
Zakay-Rones et al. (1995)	Patients with flu symptoms, 5–56 years old	Sambucol^®^ 15 ml × 2 daily for 3 days for children and 15 ml × 4 daily for 3 days for adults	Placebo	Persistence of fever was significantly shorter in the treatment group. Improvement and complete cure took significantly longer in the placebo group.
Israel	Recruited by GP			
RCT	Participants: 27 (15:12)			

Evaluation of the quality of the studies is given in Table 5. One study was slightly unclear about recruitment. In conclusion all studies are small, but apparently well conducted.

**Table 5:** **Quality of the studies of efficacy of *Sambucus nigra***.

**Paper**	**T/P/B^1^**	**Random sequence generation**	**Allocation concealment**	**Blinding of patient and personnel**	**Recruitment**	**Quality**	**Pos/neg result**
Kong, 2009	T	+	+	+	?	High	Pos
Zakay-Rones et al., 2004	T	+	+	+	GP	High	Pos
Zakay-Rones et al., 1995	T	+	+	+	Dispensary in kibbutz	High	Pos

1 Treatment, prevention, both.

The studies have evaluated efficacy from one or more of the following criteria:

- Relief of symptoms
- Duration of illness
- Additional painkillers or other pharmaceuticals used
- Duration of fever
- Serological analyses/virus isolation

All three studies gave a positive result of the use of *S. nigra* against URTI, in the form of faster recovery.

## Discussion

The results from the human and animal studies of *Echinacea* sp. are not sufficient to conclude on the safety in pregnancy. The human study (Gallo et al., 2000) was prospective with respect to time of birth but the women had used various echinacea preparations in various doses for various durations. The preparations might contain any or all of the three most common *Echinacea* species. It is reassuring that no significant differences were found between the two groups in the study with respect to the outcomes studied, however with only 206 participants taking echinacea, less frequent unwanted effects cannot be ruled out. Of the 206 women who had used echinacea, 112 had done so during the first trimester. With an estimated baseline risk of major malformations of 3.5%, a study power ≥80% and an Alpha Error Level of 5% Gallo et al. (Gallo et al., 2000) could exclude a 3.5 times increase in baseline risk of major malformations (DSS Research, 2012). No firm conclusions on the risk of spontaneous abortions can be drawn from the animal studies. The extrapolation of the fetal resorption noticed in mice (Chow et al., 2006) to risk of abortion in humans lacks further documentation and with only three mice in the treatment and three in the control group the study gives a very weak indication of risk. The contradictive results on angiogenesis in only eight mice (Barcz et al., 2007) are difficult to relate to human conditions. The authors recommend that health care personnel omit communicating data from animal studies or other unconfirmed hypothesis to pregnant women.

No documentation is available about the safety of use of *Sambucus nigra* during pregnancy, and the extent of such use is only reported in Norway (Nordeng and Havnen, 2004). One study (Tsui et al., 2001) from the USA mentions use, but does not quantify it. This apparently limited use might explain the lack of safety data.

A case report is often the first indication of a risk, but none were located on either of the herbs. The lack of case reports can be due to the lack of side effects to report or because health care personnel do not ask pregnant women about herb use (Holst et al., 2009b) or the women do not report it and connections are not made. It is important that doctors or midwives in the antenatal care ask pregnant women about herb use and that they do it in a non-judgmental way. This non-judgmental approach is essential to get a reliable answer, and thereby acquiring more documentation.

To do a benefit-risk evaluation of a treatment there is a need for efficacy-data in addition to safety documentation. A method for benefit-risk analysis evaluation is given in the ICH guideline E2C from the EMA (European Medicines Agency, 2013c).

The studies evaluate efficacy from different criteria making comparison difficult. For echinacea only two criteria; “Virus count in nasal secretion” and “Infection rate after viral challenge” are objective. The others are according to patients' experience. The main findings in the studies are concerned with duration or severity of the cold but no firm conclusions can be drawn. This is in accordance with the latest Cochrane review last edited in 2009 (including articles up to 2005) which concludes that some preparations based on the herb of *E. purpurea* might be effective in reduction of duration and severity of a cold whereas others do not (Linde et al., 2006). An important disadvantage of the studies is that it is difficult to compare amounts of active substances given in the various studies due to different ways of defining them (see Table 2, column “Intervention”). The quality of the studies is variable (see Table 3). Schoop et al (Schoop et al., 2006) have published an open study, so lack of randomization is obvious, but the paper by Turner et al. from 2000 (Turner et al., 2005) lacks the information needed to evaluate how randomization and blinding is performed. Other studies are unclear with respect to randomization or blinding or both. Schulten et al. (2001) have performed their study with employees of the manufacturer of the study product which could probably bias the result. Of the seven studies with risk of bias, four showed negative results and three positive. The distribution of positive and negative results are also even over time. This indicates that the efficacy of *Echinacea* sp. is dubious based on the identified studies and combined with the lack of safety documentation the conclusion is that the products should not be recommended to pregnant women.

The documentation of efficacy of *Sambucus nigra* is also sparse with only three studies. The main criterion for evaluation of efficacy in those is “Relief of symptoms” but various others are included. Only the serological analyses or virus isolation performed in two studies (Zakay-Rones et al., 1995, 2004) are objective. The others are according to patients' experience. The three studies all conclude that the use of *Sambucus nigra* will lead to faster recovery from influenza. However, only 77 patients were given the treatment, therefore no firm conclusions can be drawn about efficacy. As there is also a lack of safety documentation *Sambucus nigra* should not be recommended to pregnant women.

It is possible that more studies on efficacy of *Echinacea* sp. are available in German or French but this review has only taken into consideration studies published in English or in Scandinavian languages (none found). This may be a limitation but there are good reasons to believe that if positive results were discovered, they would be published in English to reach as wide an audience as possible. Of note, we did not have access to the trademark-products used in the studies included in this review and consequently could not evaluate their legal status and their compliment with the official plant monographs (Blumenthal et al., 2000; European Medicines Agency, 2008, 2009, 2010, 2012).

## Conclusion

Documentation of efficacy against URTI and safety in pregnancy is insufficient to permit a benefit-risk evaluation of *Echinacea* sp. or *Sambucus nigra* against URTI in pregnancy. Health care personnel should therefore not advice pregnant women to use those herbs. The lack of data is not in itself an indication of a substantial risk to the fetus. Women who have already used the herbs in pregnancy should be told that the recommendation not to use the herbs is given due to lack of safety data, and not due to data showing adverse effects during pregnancy. This is important to avoid unnecessary anxiety.

### Conflict of interest statement

The authors declare that the research was conducted in the absence of any commercial or financial relationships that could be construed as a potential conflict of interest.
